# Gene-Gene Associations with the Susceptibility of Kawasaki Disease and Coronary Artery Lesions

**DOI:** 10.1371/journal.pone.0143056

**Published:** 2015-11-30

**Authors:** Ho-Chang Kuo, Jen-Chieh Chang, Mindy Ming-Huey Guo, Kai-Sheng Hsieh, Deniz Yeter, Sung-Chou Li, Kuender D. Yang

**Affiliations:** 1 Department of Pediatrics, Kaohsiung Chang Gung Memorial Hospital, Kaohsiung, Taiwan; 2 Chang Gung University College of Medicine, Taoyuan, Taiwan; 3 Kawasaki Disease Center, Kaohsiung Chang Gung Memorial Hospital, Kaohsiung, Taiwan; 4 Genomic & Proteomic Core Laboratory, Department of Medical Research, Kaohsiung Chang Gung Memorial Hospital, Kaohsiung, Taiwan; 5 Department of Pediatrics, Mackay Memorial Hospital, Taipei, Taiwan; 6 Institute of Clinical Medicine, National Yang Ming University, Taipei, Taiwan; Chinese Academy of Medical Sciences, CHINA

## Abstract

Kawasaki disease (KD) is a systemic vasculitis primarily affecting children < 5 years old. Genes significantly associated with KD mostly involve cardiovascular, immune, and inflammatory responses. Recent studies have observed stronger associations for KD risk with multiple genes compared to individual genes. Therefore, we investigated whether gene combinations influenced KD susceptibility or coronary artery lesion (CAL) formation. We examined 384 single-nucleotide polymorphisms (SNPs) for 159 immune-related candidate genes in DNA samples from KD patients with CAL (n = 73), KD patients without CAL (n = 153), and cohort controls (n = 575). Individual SNPs were first assessed by univariate analysis (UVA) and multivariate analysis (MVA). We used multifactor dimensionality reduction (MDR) to examine individual SNPs in one-, two-, and three-locus best fit models. UVA identified 53 individual SNPs that were significantly associated with KD risk or CAL formation (*p* < 0.10), while 35 individual SNPs were significantly associated using MVA (*p* ≤ 0.05). Significant associations in MDR analysis were only observed for the two-locus models after permutation testing (*p* ≤ 0.05). In logistic regression, combined possession of *PDE2A* (rs341058) and *CYFIP2* (rs767007) significantly increased KD susceptibility (OR = 3.54; *p* = 4.14 x 10^−7^), while combinations of *LOC100133214* (rs2517892) and *IL2RA* (rs3118470) significantly increased the risk of CAL in KD patients (OR = 5.35; *p* = 7.46 x 10^−5^). Our results suggest varying gene-gene associations respectively predispose individuals to KD risk or its complications of CAL.

## Introduction

Kawasaki disease (KD) is an acute febrile illness that predominately affects children under 5 years of age. KD is characterized by the development of an autoimmune-like vasculitis involving the small- to medium-sized arteries, and has a predilection for the coronary arteries. KD patients present with marked elevation of various circulating immune and inflammatory cells, which infiltrate pass activated endothelial cells and into the vascular wall. As a result, up to 25 to 30% of untreated KD patients develop coronary artery lesions (CAL) including coronary artery dilation, aneurysms, or fistula formation. In rare cases, cardiac failure or thrombosis can occur and may result in sudden death (1 to 2%)[1, 2]. Therefore, prompt detection of acute KD is crucial and must be followed by timely treatment before the 10th day after disease onset, as delayed treatment with intravenous immunoglobulin (IVIG) is significantly associated with an increased risk of CAL formation in KD patients. In addition, approximately 10% of all KD patients do not respond to IVIG treatment, which is significantly associated with a higher risk of CAL formation[3].

To fulfill a diagnosis of KD, patients must develop a high-grade fever lasting longer than five days that does not respond to either antibiotics or antipyretics, in addition to four out of the following five principal diagnostic features: 1) conjunctivitis; 2) changes in the extremities; 3) oral changes; 4) polymorphous rash; and 5) cervical lymphadenitis[4].

The cause of KD remains unknown despite several decades of extensive international investigation. Genetic investigations are now primarily used to identify pathways involved in KD so that its cause may ultimately be discovered. This has led to a wealth of reports largely regarding single-nucleotide polymorphisms (SNP) that are associated with cardiovascular, inflammatory, or immune responses. However, numerous genetic findings are later found to be inconsistent or conflicting in KD upon replication.

The genetic propensity to develop KD seems to be greatly influenced by ethnicity. Not only are Asian children 10 to 20 times more likely to develop KD when compared to other ethnic groups[5], the genes associated with KD and the degree of their expression appears to differ among varying ethnic populations, including ethnic Han Chinese, Korean, or Japanese children[6]. In response to this situation, genome-wide association studies (GWAS) and their meta-analyses are now employed in differing ethnic populations to determine the statistical significance of genetic associations. This has led to the apparent confirmation of several susceptibility loci in KD, including SNPs for the *FCGR2A*, *BLK*, *CD40*, *ITPKC*, and *CASP3* genes[7–9]. However, these are fairly modest genetic findings of an increased risk for KD susceptibility and do not reveal any primary genes that are involved in the development of KD.

Several authors have recently begun to investigate potential gene-gene interactions in KD patients, and have found that gene-gene associations may have a greater predictive value for the development and prognosis of KD when compared to individual SNPs alone. For example, prior studies have found that patients who possess the susceptibility allele SNPs for both *ITPKC* and *CASP3* were more significantly associated with IVIG resistance when compared with those with only one susceptible SNP[10, 11]. Therefore, we examined 159 immune-related candidate genes using a commercialized 384-SNP multiplex microarray to identify potential gene-gene interactions associated with KD risk or subsequent CAL formation. In addition, we also collected the plasma levels for certain inflammatory and immune markers in KD patients to correlate the functional effect of significantly identified gene-gene associations.

## Materials and Methods

### Study participants

Our study received the approve consent procedure from the Institutional Review Board of the Chang Gung Memorial Hospital in Taiwan. We collected blood samples after written informed consent had been obtained from either the parents or guardians. For patients with KD, blood samples were collected before IVIG treatment. Our study participants included Taiwanese children who completely fulfilled the diagnostic criteria for KD according to the American Heart Association guidelines and were admitted to the Kaohsiung Chang Gung Memorial Hospital for IVIG treatment between 2001 and 2006. Previously, we had investigated this sample of KD patients for biomarkers of IVIG treatment resistance[12] and CAL formation[13].

KD patients were treated with a single dose of IVIG (2 g/kg) during a 12-hour period. Aspirin was administered until all of the signs of inflammation resolved and CAL regressed as detected with two-dimensional (2D) echocardiography. Principle clinical features of KD that occur during the acute stage of the illness were recorded and coded for analysis. Each patient with KD underwent 2D echocardiography of the coronary arteries before treatment with IVIG. Two subsequent echocardiograms were performed within the 4 weeks following IVIG treatment. CAL were defined as the internal diameter of the coronary artery being at least 3 mm for KD patients aged 0 to 5 years (4 mm in patients > 5 years of age) or if the internal diameter of a coronary artery segment was at least 1.5 times larger than an adjacent segment as detected by echocardiogram[14].

According to PASS 2008 Statistical Software (Utah, USA), we initially estimated the sample size at 59 patients with CAL formation in KD subjects based on a study power of 0.8, with significance at < 0.05 for two-sided alternative hypothesis, the 25 to 30% of untreated KD patients may develop CAL, and 10% of all KD patients do not respond to IVIG whose risk of CAL formation are very high[3]. We finally included 73 children with CAL formation (32.3%) in the study from 226 KD subjects. 575 control subjects for KD susceptibility were obtained from our previous investigations of a Taiwanese birth cohort examining gene-gene interactions with IgE production until the sixth year of age[15, 16]. Our cohort controls were confirmed to have no history of KD, while more than 90% of KD cases occur by the sixth year of age. We used KD patients without CAL as controls to investigate the risk of CAL formation.

### Collection of plasma and DNA from peripheral blood samples

We collected blood samples from KD patients and cohort controls in heparin tubes. Plasma was prepared from the blood samples by centrifuge at 3,000 rpm for 10 minutes and then stored in six aliquots. DNA samples were harvested from total leukocytes in the plasma using cell lyses buffer according to instructions from the manufacturer (GENETRA DNA extraction kit, Minneapolis, MN).

### Amplification of genomic DNA for oligonucleotide-based 384-SNP microarray

The criteria for selection of 384 SNPs of 159 candidate genes were mainly based on several previously reports as described on our previous publication[16]. These SNPs were preliminarily screened via a proprietary algorithm of Illumina (San Diego, CA, USA) according to their performance on the Illumina platform. After excluding the SNPs containing minor allele frequency less than 5% in Chinese population, we selected 91 SNPs in 44 innate genes, 102 SNPs in 37 adaptive genes, and 191 SNPs in 78 oxidative stress and remodeling genes for the 384 customized SNPs design on different chromosomes. In total, 384 SNPs from 159 candidate genes with representative SNPs in the NCBI Genome Build 36.3 (dbSNP build 129) were genotyped with the Illumina BeadStation 500GX. DNA samples were quantified with a PicoGreen dsDNA Quantitation Kit (Molecular Probes, Eugene, OR, USA). After DNA quantification, DNA samples were adjusted to 50 ng/μL in a TE buffer (Tris-HCl, 10 mM, EDTA 1 mM, pH 8.0) for PCR amplification in Oligo Pool All (OPA), which contained a set of all the primers for every individual SNP. The PCR products were hybridized and then analyzed using BeadStudio software (version 2.1.10) for genotyping. We conducted the genotyping in accordance with the manufacturer's recommendations and as outlined in our previous investigations of the allergy cohort[15, 16].

### Verification of 384-SNP microarray data accuracy through repeat measurements and concurrent experiments of restriction fragment length polymorphisms

To further verify the accuracy of the 384-SNP multiplex microarray, we performed a parallel genotyping experiment to validate significantly identified gene-gene associations for the *PDE2A* (rs341058), *CYFIP2* (rs767007), and *IL2RA* (rs3118470) SNPs. Restriction fragment length polymorphism methods were conducted using DNA samples (n = 98) from our KD patient sample. The *LOC100133214* (rs2517892) SNP was respectively validated via pyrosequencing assay using a PyroMark Q24 instrument (Qiagen, Valencia, CA, USA). The genotyping accuracy for the *PDE2A*, *CYPIP2*, *IL2RA* and *LOC100133214* SNPs between these two methods was 100%.

### Measurement of plasma cytokines in KD patients

A total of n = 73 KD patients, including n = 35 with CAL formation, from our sample were further enrolled to examine cytokines levels in the illness. Plasma concentrations of IL-2, IL-3, IL-4, IL-5, IL-6, IL-8, IL-10, IL-17A, and IFN-γ in KD patients were assessed using the Upstate Beadlyte Human Cytokine Beadmates System (Upstate Group, Inc.) in association to high-risk genotypes for our identified gene-gene combinations. In summary, we mixed 50 μl plasma samples with multiplexed antibody-conjugated beads, which were then subjected to a multi-channel detection of their bead-array. Acquired fluorescence data was assessed by MasterPlexTM QT software (Ver. 1.2; MiraiBio, Inc.). We determined the calibration of cytokine concentrations in KD patients through the interpolation of a series of well-known standard samples in accordance to the manufacturer’s recommendation. The plasma levels of TGF-β1 in KD patients were determined with ELISA using a commercial kit (R&D Systems). This study method was modified from our previous report examining risk factors for CAL formation in KD patients.

### Data analysis and statistics

Individual SNPs were initially examined among KD patients and cohort controls by univariate analysis (UVA) to identify associations with the risk of KD or CAL formation. We then used multivariate analysis (MVA) to investigate the respective allele combinations for individual SNPs among KD patients and controls in association with the development of KD or CAL formation. The variables for MVA consisted of a dominant (AA/AB vs. BB), co-dominant (AB vs. AA/BB), recessive (BB vs. AB/AA), and additive genotype (number of A or B alleles). Using these variables, we determined the best fitting genetic model for the allele variations of each individual SNP. For our study purposes, a *p*-value < 0.10 was considered to be statistically significant in UVA, while we considered a *p*-value ≤ 0.05 to be statistically significant in MVA.

To investigate potential gene-gene interactions associated with either KD risk or CAL formation, we used a multifactor dimensionality reduction (MDR) method to examine each individual SNP in a one-, two-, and three-locus best fit model. Statistically significant best fit models were further analyzed in logistic regression. We classified statistically significant gene-gene associations into high- and low-risk genotypes using the varying combinations of alleles for identified SNPs. High- and low-risk groups were then analyzed in the Chi-square test. We conducted our analysis with MDR software (version 1.1.0), which is a freely available program that is part of a collaborative open-source project (sourceforge.net/projects/mdr/).

Several methods to correct for multiple testing are useful for candidate gene studies, especially for GWAS. The Bonferroni correction and permutation are common adjustments. In contrast to the Bonferroni correction, permutation tests can give the optimal exact threshold and are considered the gold standard in multiple testing adjustments for genetic association studies[17]. The MDR analysis incorporates a cross-validation/permutation procedure to minimize the rate of false positive findings that may otherwise result from tests involving multiple variables or comparisons[18]. In this study, the predictive performance of the best model is then assessed through 20-fold cross-validation and its significance determined through Monte Carlo permutation testing[19, 20]. Calculate *p*-value by comparing where the observed test statistic value lies in the permuted distributed of test statistics as following description. To identify the best fit models, we performed 20 cross-validation runs of permutation testing to calculate the cross-validation consistency (CVC) and prediction errors for every pooled combination of individual SNPs. Pooled SNPs were chosen in a cross-validation run when they presented with the highest training-balanced accuracy [(Sensitivity + Specificity) / 2], while the CVC involved the number of times a group of pooled SNPs was selected in a cross-validation run. Individual groups of pooled SNPs with the highest CVC following 20 cross-validation runs were selected as the best fit for the one-, two-, and three-locus models. Statistical significance was determined by comparing average the prediction errors from our observed data to the average prediction errors under the null hypothesis of no association, which we derived empirically from 10,000 permutations. The null hypothesis was rejected when the *p*-value derived from our permutation testing was ≤ 0.05.

## Results

### Clinical features of KD patients

A total of n = 801 cases were enrolled in our current investigation, including n = 226 patients with KD and n = 575 cohort controls. There were n = 230 female (40.0%) and n = 345 male (60.0%) subjects in the control group. Among the KD group, males accounted for n = 153 cases (67.6%) and females accounted for n = 73 cases (33.4%). All of our patients with KD presented with fever (100%), while most of our KD patients also presented with conjunctivitis (96.5%), fissured lips (92.9%), a strawberry tongue (87.6%), changes in the extremities (86.3%), and polymorphous skin rashes (88.5%). Approximately half of these KD patients presented with lymphadenopathy. For children in the KD group, n = 73 cases (32.3%) developed CAL formation.

### Excluded SNPs in candidate genes

A total of 345 SNPs assessed in DNA samples from KD patients (n = 226) and cohort controls (n = 575) were included in our final analysis. We excluded a total of 39 SNPs from our analyses that possessed either a call rate < 90% or were beyond the Hardy-Weinberg equilibrium (HWE), as shown in S1 Table. Most of our excluded genes with an SNP distribution beyond the HWE were located in HLA-DR regions that have been previously reported to be distributed beyond the HWE[15].

### Univariate and multivariate analysis

We identified 31 individual SNPs for 27 genes that were significantly associated with KD susceptibility in UVA (*p* < 0.10), as shown in S2 Table. After using MVA to investigate allele variations, we identified 23 individual SNPs for 22 genes that demonstrated associations with increased KD risk in a statistically significant manner (*p* ≤ 0.05), as shown in Table 1. The majority of these best fit allele variations identified in MVA demonstrated a protective effect against KD development (60.9%). More than half of protective alleles were recessive (57.1%), while only one susceptibility allele was recessive (7.1%). The recessive T allele of an SNP for the *SPP1* gene (rs2853744) demonstrated the greatest protective effect against the development of KD (OR = 0.04; *p* = 0.028), while the dominant T allele of an SNP for the *PDGFRA* gene (rs4358459) demonstrated the highest risk of KD development (OR = 4.24; *p* = 0.027).

**Table 1:** Multivariate analysis of 345 SNPs associated with KD susceptibility in patients and cohort controls (*p* ≤ 0.05).

Gene	dbSNP	Best fitting genetic model	*p-*value	OR (95% CI)
**Innate immunity**				
*SPP1*	rs2853744	Recessive (TT vs. TG/GG)	0.028	0.04 (0.00–0.72)
*CLEC4C*	rs10845821	Recessive (TT vs. TC/CC)	0.011	0.45 (0.25–0.83)
*SPP1*	rs2728127	Additive (number of G alleles)	0.011	0.89 (0.82–0.97)
*COLEC11*	rs10210631	Dominant (AA/AG vs. GG)	0.030	1.51 (1.04–2.19)
*C5*	rs17611	Recessive (AA vs. AG/GG)	0.012	1.67 (1.12–2.49)
*CD209*	rs2287886	Dominant (AA/AG vs. GG)	0.002	3.50 (1.58–7.76)
**Adaptive immunity**				
*HLA-DQA1*	rs2040410	Recessive (AA vs. AG/GG)	0.035	0.12 (0.02–0.86)
*TBX21*	rs2240017	Co-dominant (CG vs. CC/GG)	6.92*10^−5^	0.37 (0.23–0.61)
*TAP1*	rs2071541	Recessive (TT vs. TC/CC)	0.011	0.59 (0.40–0.89)
*LY75*	rs2042772	Recessive (TT vs. TC/CC)	0.031	0.66 (0.46–0.96)
*IL13*	rs1800925	Additive (number of C alleles)	0.003	0.94 (0.91–0.98)
*HLA-DPB1*	rs3097671	Additive (number of G alleles)	2.10*10^−4^	1.10 (1.05–1.16)
*IL5RA*	rs340833	Co-dominant (AG vs. AA/GG)	0.014	1.61 (1.10–2.34)
**Stress and response**				
*ADAM33*	rs3918400	Recessive (TT vs. TC/CC)	0.018	0.24 (0.07–0.78)
*ELF5*	rs836145	Recessive (TT vs. TG/GG)	0.009	0.54 (0.34–0.86)
*PIK3CD*	rs11121484	Co-dominant (TC vs. TT/CC)	0.043	0.57 (0.33–0.98)
*PDE2A*	rs341058	Recessive (AA vs. AG/GG)	0.040	0.68 (0.47–0.98)
*CYFIP2*	rs767007	Additive (number of G alleles)	0.042	0.96 (0.92–1.00)
*PEX6*	rs2274514	Additive (number of G alleles)	0.005	0.98 (0.97–0.99)
*SELP*	rs6128	Additive (number of G alleles)	0.016	1.02 (1.00–1.04)
*ADRB2*	rs1042713	Co-dominant (AG vs. AA/GG)	0.014	1.59 (1.10–2.29)
*PIM1*	rs262918	Dominant (TT/TC vs. CC)	0.021	2.07 (1.11–3.83)
*PDGFRA*	rs4358459	Dominant (TT/TG vs. GG)	0.027	4.24 (1.18–15.31)

We identified 22 individual SNPs for 21 genes that were significantly associated with CAL formation in KD patients using UVA (*p* < 0.10), as shown in S3 Table. After using MVA to investigate allele variations, 12 SNPs for 12 genes that were significantly associated with CAL formation in KD patients (*p* ≤ 0.05), as shown in Table 2. The majority of these allele variations were significantly associated with an increased risk of CAL formation in patients with KD (75.0%). Half of the identified susceptibility alleles were co-dominant (50.0%), while only one protective allele was co-dominant (25.0%). The dominant T allele of an SNP for the *IL4* gene (rs2243250) demonstrated the greatest protective effect against the development of CAL in KD patients (OR = 0.03; *p* = 0.006), while the dominant A allele of an SNP for the *CD14* gene (rs2569190) demonstrated the highest risk of CAL formation in KD patients (OR = 5.72; *p* = 0.005).

**Table 2:** Multivariate analysis of 345 SNPs associated with CAL formation in KD patients (*p* ≤ 0.05).

Gene	dbSNP	Best fitting genetic model	*p-*value	OR (95% CI)
**Innate immunity**				
*CD209*	rs12611071	Recessive (AA vs. AC/CC)	0.007	0.20 (0.06–0.64)
*NOD2*	rs2111235	Additive (number of C alleles)	0.006	1.28 (1.07–1.53)
*CLEC2D*	rs1863873	Co-dominant (TC vs. TT/CC)	0.032	2.36 (1.08–5.16)
*CXCL10*	rs867562	Co-dominant (AG vs. AA/GG)	0.002	3.41 (1.55–7.49)
*CCL24*	rs2302004	Co-dominant (TC vs. TT/CC)	0.001	3.64 (1.64–8.07)
*CD14*	rs2569190	Dominant (AA/AG vs. GG)	0.005	5.72 (1.67–19.60)
**Adaptive immunity**				
*IL4*	rs2243250	Dominant (TT/TC vs. CC)	0.006	0.03 (0.00–0.38)
*CD80*	rs1485332	Additive (number of G alleles)	0.001	1.22 (1.09–1.38)
*MS4A2*	rs2583476	Co-dominant (TC vs. TT/CC)	0.001	3.81 (1.69–8.57)
**Stress and response**				
*LTC4S*	rs730012	Co-dominant (AC vs. AA/CC)	0.027	0.29 (0.10–0.87)
*ADAM33*	rs3918400	Additive (number of C alleles)	0.015	0.91 (0.84–0.98)
*EHF*	rs286902	Recessive (AA vs. AG/GG)	0.003	3.31 (1.51–7.26)

### Multifactor dimensionality reduction analysis

We identified pooled SNPs with the highest training-balanced accuracy in one-, two-, and three-way best fit models. Significant associations were only observed with two-locus models after permutation testing (*p* ≤ 0.05). For the increased risk of KD susceptibility, two SNPs of *PDE2A* (rs341058) and *CYFIP2* (rs767007) revealed the highest training-balanced accuracy average (53.7%) in the two-way model (Table 3), with an OR = 3.54 in logistic regression (*p* = 4.14 x 10^−7^). For the subsequent risk of CAL formation in KD patients, two SNPs of *LOC100133214* (rs2517892) and *IL2RA* (rs3118470) revealed the highest average training-balanced accuracy (53.6%) in the two-way model (Table 4), with an OR = 5.35 in logistic regression (*p* = 7.46 x 10^−5^).

**Table 3:** Best fit results using multifactor dimensionality reduction analysis of one-, two-, and three-locus models for KD susceptibility in patients and cohort controls.

Gene (polymorphism)	^a^ Average testing balanced accuracy	^b^ Average cross validation consistency	^c^ p-value
TBX21 (rs2240017)	50.32%	12/20	0.868
PDE2A (rs341058) and CYFIP2 (rs767007)	53.73%	17/20	0.021**
STAT3 (rs1026916), CLEC7A (rs2078178), and PSMB8 (rs3763364)	43.24%	6/20	0.999

^a^ Average testing balanced accuracy is the accuracy of classifications for cases and controls in the testing dataset (one-twentieth of the data) calculated as (Sensitivity+Specificity)/2.

^b^ Average cross validation consistency is the number of times the model was selected as the best model after 20 cross-validation runs.

^c^ Significance of accuracy (empirical p-value based on 10,000 permutations).

** p-value ≤ 0.05

**Table 4:** Best fit results using multifactor dimensionality reduction analysis of one-, two-, and three-locus models for CAL formation in KD patients.

Gene (polymorphism)	^a^ Average testing balanced accuracy	^b^ Average cross validation consistency	^c^ p-value
LY75 (rs2042772)	45.59%	12/20	0.588
LOC100133214 (rs2517892) and IL2RA (rs3118470)	53.60%	13/20	0.021**
FGF1 (rs249923), CLEC2D (rs1863873), and CCL2 (rs2857656)	43.42%	5/20	0.942

^a^ Average testing balanced accuracy is the accuracy of classifications for cases and controls in the testing dataset (one-twentieth of the data) calculated as (Sensitivity+Specificity)/2.

^b^ Average cross validation consistency is the number of times the model was selected as the best model after 20 cross-validation runs.

^c^ Significance of accuracy (empirical p-value based on 10,000 permutations).

** p-value ≤ 0.05

**Fig 1:**
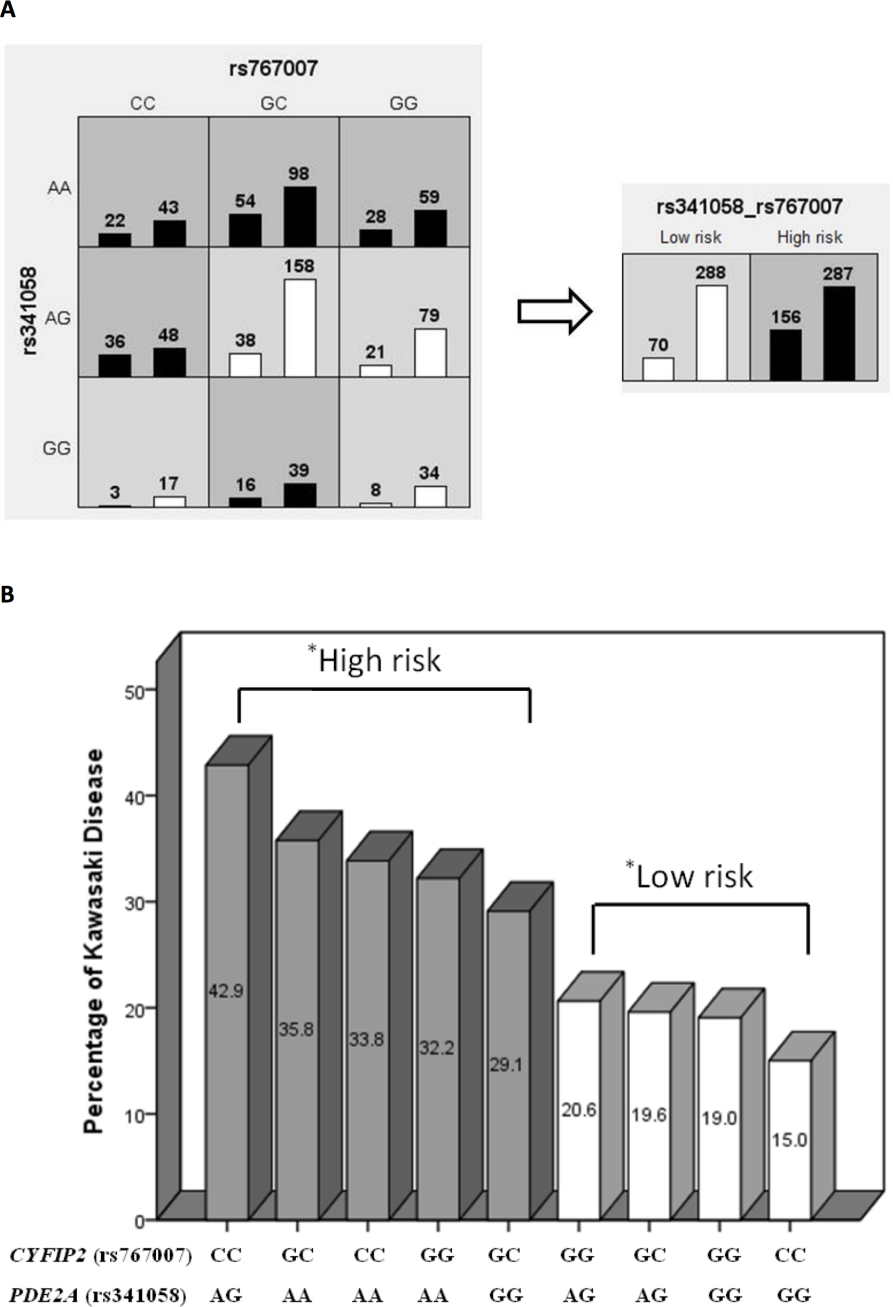
*PDE2A* (rs341058) and *CYFIP2* (rs767007) gene-gene interaction in a two-way mode of MDR analysis. The interaction of *PDE2A* and *CYFIP2* was significantly associated with increased KD risk in logistic regression of our MDR results from KD patients (n = 226) and cohort controls (n = 575), with an odds ratio of 3.54 (95% CI: 2.17–5.78) and a *p*-value of 4.14 x 10^−7^. (A) MDR classified the nine interactive items of allele combinations into high- or low-risk KD groups, which were significantly different in our further analysis using the Chi-square test (p = 9.71 x 10–7). (B).

**Fig 2:**
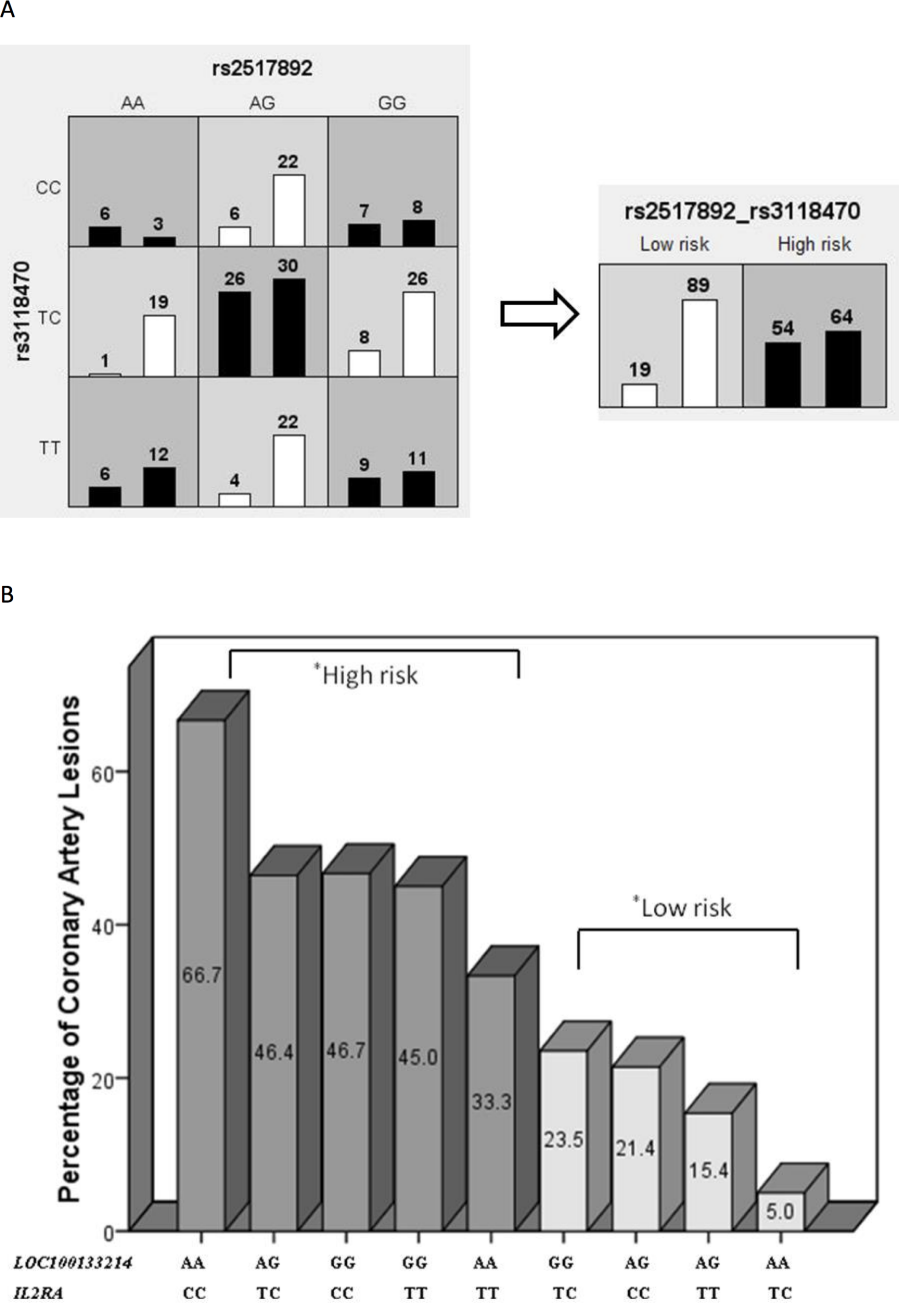
*LOC100133214* (rs2517892) and *IL2RA* (rs3118470) gene-gene interaction in a 2-way mode of MDR analysis. The interaction of *LOC100133214* and *IL2RA* was significantly associated with a higher risk of CAL formation using logistic regression of our MDR results from KD patients with CAL (n = 73) and KD patients without CAL (n = 153), with an odds ratio of 5.35 (95% CI: 2.33–12.25) and a *p*-value of 7.46 x 10^−5^. (A) MDR classified the nine interactive items into high- or low-risk CAL groups, which were significantly different in our further analysis using the Chi-square test (*p* = 3.36 x 10^−6^). (B).

The MDR results of these significant associations in the two-locus model were further analyzed using the Chi-square test. As shown in Fig 1A and 1B, our classification of varying allele combinations for the *PDE2A* and *CYFIP2* SNPs identified in MDR revealed four low-risk genotypes (n = 358) and five high-risk genotypes (n = 443) that significantly differed between the KD group and cohort controls (*p* = 9.71 x 10^−7^). As shown in Fig 2A and 2B, our classification of varying allele combinations for the *LOC100133214* and *IL2RA* SNPs revealed four low-risk genotypes (n = 118) and five high-risk genotypes (n = 108) that differed significantly between KD patients with subsequent CAL formation and KD patients without CAL (*p* = 3.36 x 10^−6^).

Among the nine allele classifications, we observed that combinations of the AG allele for *PDE2A* and the CC allele for *CYFIP2* conferred the highest risk of KD susceptibility (42.9%), while combinations of the CC allele for *CYFIP2* and the GG allele for *PDE2A* conferred the lowest risk (15.0%). No significant associations were observed between these genotype combinations of the *CYFIP2* and *PDE2A* SNPs with the development of either CAL formation or IVIG resistance in KD (*p* < 0.10). We also found that combinations of the AA allele for *LOC100133214* and the CC allele for *IL2RA* conferred the highest risk of CAL formation in KD patients (66.7%), while combinations of the TC allele of *IL2RA* and the AA allele of *LOC100133214* conferred the lowest risk (5.0%). We did not observe any significant associations for these genotypes with either KD risk or the development of IVIG resistance (*p* < 0.10).

**Fig 3:**
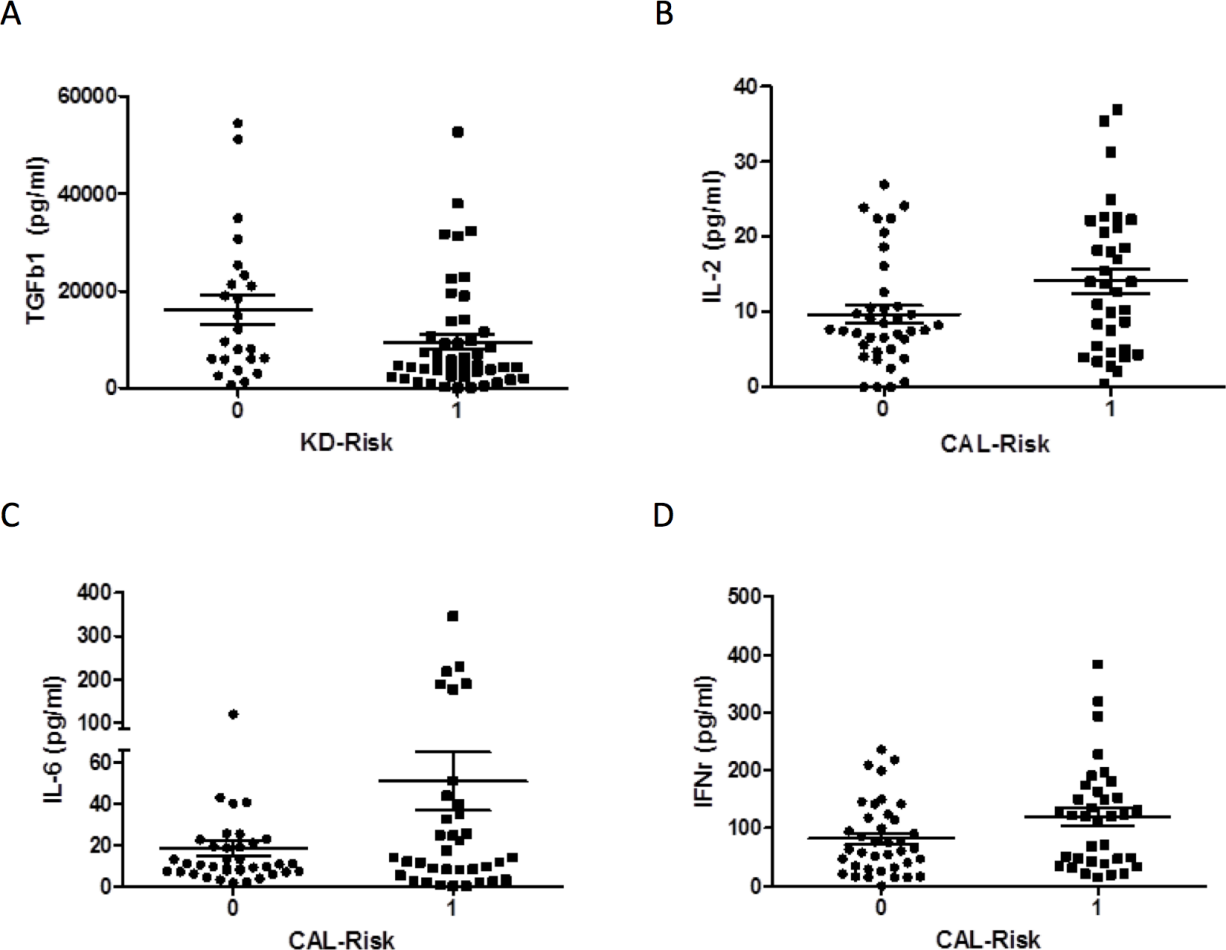
Comparison cytokines levels between KD patients with high-risk genotypes and low-risk genotypes. KD patients possessing the high-risk (KD risk: 1) *PDE2A* (rs341058) and *CYFIP2* (rs767007) genotypes of KD susceptibility (n = 49) presented with significantly lower plasma levels of TGF-β1 (9489 ± 1605 vs. 16133 ± 3015) compared to KD patients in the low-risk group (KD risk: 0, n = 24), with an odds ratio of 0.59 (*p* = 0.036). (A) KD patients possessing the high-risk *LOC100133214* (rs2517892) and *IL2RA* (rs3118470) genotypes of CAL formation (CAL risk: 1, n = 35) presented with significantly elevated plasma levels of IL-2 (14.1 ± 1.6 vs. 9.6 ± 1.2) compared to KD patients in the low-risk group (CAL risk: 0, n = 38), with an odds ratio of 1.47 (*p* = 0.028). (B) KD patients possessing the high-risk *LOC100133214* (rs2517892) and *IL2RA* (rs3118470) genotypes of CAL formation (CAL risk: 1, n = 35) presented with significantly elevated plasma levels of IL-6 (51.0 ± 14.3 vs. 18.4 ± 3.7) compared to KD patients in the low-risk group (CAL risk: 0, n = 38), with an odds ratio of 2.77 (*p* = 0.033). (C) KD patients possessing the high-risk *LOC100133214* (rs2517892) and *IL2RA* (rs3118470) genotypes of CAL formation (CAL risk: 1, n = 35) presented with significantly elevated plasma levels of IFN-γ (119.2 ± 15.2 vs. 81.8 ± 10.1) compared to KD patients in the low-risk group (CAL risk: 0, n = 38), with an odds ratio of 1.46 (*p* = 0.041). (D).

High-risk allele combinations of the *PDE2A* and *CYFIP2* SNPs accounted for 67.1% of our KD cases. As shown in Fig 3A, we observed that these high-risk genotypes in KD patients were significantly associated with reduced plasma levels of TGF-β1 (9489 ± 1605 vs. 16133 ± 3015 pg/ml; *p* = 0.036) compared to KD patients in the low-risk group. High-risk allele combinations of the *LOC100133214* and *IL2RA* SNPs accounted for 47.9% of our KD cases. We found significantly elevated plasma levels of interleukin (IL)-2 (14.1 ± 1.6 vs. 9.6 ± 1.2 pg/ml; *p* = 0.028), IL-6 (51.0 ± 14.3 vs. 18.4 ± 3.7 pg/ml; *p* = 0.033), and Interferon-γ (119.2 ± 15.2 vs. 81.8 ± 10.1 pg/ml; *p* = 0.041) in KD patients with the high-risk genotypes of CAL formation compared to KD patients in the low-risk CAL formation genotype group, as shown in Fig 3B, 3C and 3D. No significant differences were found between high- and low-risk genotypes in KD patients with levels of IL-3, IL-4, IL-5, IL-10, or IL-17A.

## Discussion

To investigate gene-gene associations for the development of KD or its outcomes, we used an MDR method of analysis to establish the best fit models after repeated permutation testing for either individual or multiple genes. The cross-validation/permutation procedure incorporated in our MDR analysis minimizes false positive rates that result from multiple comparisons. This method of MDR analysis is able to identify a high order of evidence for gene-gene interactions in diseases when there is a lack of any primary individual genes that influence susceptibility[21], including autoimmune disorders and certain malignancies[22]. Therefore, MDR is of potential value in genetic studies of KD, which lacks a primary susceptibility marker. Numerous genes to date have been found to be associated with the development or prognosis of KD, which probably reflects the multi-genetic nature of the disease. Our approach using MDR analysis more closely matches the seemingly complex nature of genetics in KD and allows us to examine genes in a more unbiased manner, by taking into account the effect of gene-gene interactions, as opposed to the effect of one candidate gene alone. As reported in previous studies, we observed much stronger associations with KD and its outcomes for multiple genes compared to individual genes. We found that the two-locus models were significantly associated with either the development of KD or the subsequent formation of CAL (*p* ≤ 0.05), although no statistically significant associations for any of the individual genes in our one-locus model after repeated permutation testing. This was seen as a surprising development given the fact that we identified allele variations in nearly three dozen individual SNPs that were significantly associated with KD risk or CAL formation.

We also observed that varying alleles of individual SNPs or gene-gene combinations were associated with either the development of KD or its subsequent complications. We also found that KD patients who possessed the high-risk genotypes of our identified gene-gene associations also had significantly different levels of the immune and inflammatory markers that were tested in this study. Lastly, our analyses using an MDR method yielded the most significant results with the lowest *p*-value compared to our analyses using univariate or multivariate methods.

In our initial investigations using UVA and MVA, we found several dozen individual SNPs and allele variations that were significantly associated with either an increased risk of KD susceptibility or the formation of CAL. Among our results, we found that the dominant A allele of an SNP for the *DC-SIGN* (*CD209*) promoter gene (rs2287886) was significantly associated with an increased risk of KD susceptibility (OR = 3.50; *p* = 0.002). Previously, Portman *et al*. reported that the major A allele of the rs2287886 SNP for the *CD209* gene was significantly associated with IVIG-treatment resistance during acute KD among Asian children in a US population (OR = 1.76; *p* = 0.04)[23]. The authors did not find a similar association among either Caucasian or Hispanic children, for whom the A allele was only of minor frequency. We recently reported that haplotypes of *CD209* polymorphisms were significantly associated with an increased risk of KD susceptibility in Taiwanese children[24], which included the major A allele of the rs2287886 SNP (OR = 1.61; *p* = 0.0002). However, we did not observe significant associations between any polymorphisms of *CD209* polymorphisms and IVIG-treatment response for Taiwanese children with KD.

In addition, we found that KD patients with the dominant A allele of an SNP for *CD14* (rs2569190) were at greatest risk for the development of CAL formation (OR = 5.76, *p* = 0.005). Previously, an investigation in Japan reported that KD patients with the T allele of the *CD14* C(−159)T polymorphism (rs2569190) were significantly more likely to develop CAL (OR = 2.20; *p* < 0.05), although no associations were found with KD susceptibility[25]. We also observed that KD patients with the dominant T allele of an SNP for the *IL-4* gene (rs2243250) had the lowest risk of developing CAL formation (OR = 0.03; *p* = 0.006). Previously, Burns *et al*. reported the significant asymmetrical transmission of alleles for the *IL-4* C(-589)T polymorphism (rs2243250) from parents to their children with KD (*p* = 0.05) in a US population, although no association was observed with CAL formation[26]. However, SNPs of the *IL-4* gene have not been found to be associated with KD susceptibility or subsequent CAL formation among children in Taiwan[27, 28].

In our two-locus model, combined possession of SNPs for the *PDE2A* (rs341058) and the *CYFIP2* (rs767007) gene were significantly associated with increased KD susceptibility in logistic regression (OR = 3.54; *p* = 4.14 x 10^−7^), although no associations were found for the risk of CAL formation or responsiveness to IVIG treatment. Respectively, we observed that KD patients with both SNPs for *LOC100133214* (rs2517892) and *IL2RA* (rs3118470) were significantly more likely to develop CAL formation in logistic regression (OR = 5.35; *p* = 7.46 x 10^−5^), although no associations were found with the risk of developing KD or the response to IVIG treatment during the disease course. We observed even lower *p*-values in the Chi-square test for both of the identified gene-gene associations after we had separated individuals into high- and low-risk genotype groups by their respective allele combinations. KD patients with high-risk genotypes for the *PDE2A* and *CYFIP2* SNPs had significantly reduced plasma levels of TGF-β1 (OR = 0.59; *p* = 0.036) compared to the low-risk KD group, while KD patients with high-risk allele combinations for the *LOC100133214* and *IL2RA* SNPs had significantly elevated plasma IL-2 (OR = 1.47; *p* = 0.028), IL-6 (OR = 2.77; *p* = 0.033), and IFN-γ (OR = 1.46; *p* = 0.041) compared to the low-risk KD group.

In our current investigation, *PDE2A* (rs341058) and *CYFIP2* (rs767007) were the only SNPs significantly associated in UVA, MVA, and MDR analysis. To our knowledge, no other study to date has found a link between *CYFIP2* SNPs and the development or prognosis of KD, although SNPs in the gene *CYFIP2* has been found to be associated with allergic disease. In a study of 492 Mexican children, rs17599222 in the gene *CYFIP2* was found to be associated with childhood asthma[29]. Similarly, in our previous study, we found that the combination of *PDE2A* (rs341058) and *CYFIP2* (rs767007) plus an SNP of *IL-13* (rs1800925) in a three-locus model was significantly associated with increased IgE production in Taiwanese children who lacked a history of KD[15]. Recent populations studies have also found that patients with Kawasaki disease appear to have a higher subsequent risk of developing atopic dermatitis and other allergic diseases[30, 31], suggesting that both KD and allergic disease may share a similar immune response. It is possible that CYFIP2 is implicated in a common pathway shared by both KD and allergy, although more research regarding the functional effect of *CYFIP2* (rs767007) SNP mutations would be required to confirm this hypothesis.

Likewise, the link between *PDE2A* (rs341058) SNP and Kawasaki disease found in this study, is to our knowledge, a novel finding. The *PDE2A* gene encodes for phosphodiesterase 2 (PDE2), which increases the hydrolysis of cAMP after being activated by cGMP. Overexpression of PDE2 has been found in human myocytes of patients with heart failure, and blunts β-adrenergic responses via decreasing the cAMP stimulation of the L-type Ca^2+^ current[32]. This finding suggests that development of Kawasaki disease may be associated with differences in myocardial calcium current conduction; of note, previous studies have linked KD susceptibility to the C allele of the *ITPKC* SNP (rs28493229) a gene associated with the Ca^2+^/NFAT pathway[33, 34]. Tumor necrosis factor-alpha, a cytokine that is critical in the development coronary artery lesions in KD, has been found to upregulate PDE2 in cultivated human umbilical vein endothelial cells, and may play a role in increased endothelial permeability[35].

Previously, the identification of *ITPKC* susceptibility has led to a great emphasis upon the Ca^2+^/NFAT pathway in KD research. Inositol 1,4,5-triphosphate 3-kinase C (ITPKC) negatively regulates cell signaling through converting inositol 1,4,5-triphosphate (IP_3_) into its inactive state, inositol 1,3,4,5-tetrakisphosphate (IP_4_). This prevents IP_3_ from stimulating receptors upon the endoplasmic reticulum to trigger the release of Ca^2+^ from its intracellular stores[36]. The C allele of the *ITPKC* SNP (rs28493229) reduces transcription of the gene by decreasing the efficiency of RNA splicing. Therefore, *ITPKC* susceptibility in KD reduces expression of the gene and would result in the decreased inactivation of IP_3_. Subsequently, *ITPKC* susceptibility renders KD patients susceptible to the hyperactivation of Ca^2+^ signaling in the immune system, which results in an elevation of immune and inflammatory cells. As genetic studies are largely used to identify pathways involved in KD so that its environmental causes are ultimately being discovered, it is therefore concluded that the *ITPKC* susceptibility in KD sensitizes individuals to xenobiotics that induce calcium influx[33, 34].

In conclusion, our findings indicate that differing gene-gene interactions appear to be respectively associated with predisposition for the development of KD or CAL formation. Varying gene-gene interactions may account for why individual susceptibility loci in KD appear to be fairly modest or inconsistent upon larger replication and meta-analysis, while stronger associations are observed when these genes are present in combination. This suggests a fairly high order of genetic variation for KD and may reflect a multifactorial etiology in the disease process that impacts the same general pathways. The highest incidence of KD in Taiwan is 69 cases per 100,000 children under 5 years of age[37]. The major limitation of this study is that the low sample size, which reflected the difficulties inherent in recruiting patients with such a rare disease. Replication of the findings in large, well-powered independent samples is crucial if this problem is to be overcome, and will likely require a multicenter collaboration to provide strong evidence on validation of the novel gene to gene interactions discovered the susceptibility of Kawasaki disease and coronary artery lesions. This study only analyzed patients with KD in our current investigation without a formal assessment of population structure of the sampled population; there is still a possibility that the observed positive association is over-represented. These limitations should be considered in future studies.

## Supporting Information

S1 Table The SNPs excluded for the final analysis due to call rate < 90% or their genotypes’ distributions beyond Hardy Weinberg equilibrium (HWE) ≦ 0.001. (DOC)

S2 Table Association of 31 SNPs in 27 innate, adaptive and stress/response genes with CB and KD cohort UVA analysis (*p* < 0.1). (DOC)

S3 Table Association of 22 SNPs in 21 innate, adaptive and stress/response genes with/without CAL in KD cohort by UVA analysis (*p* < 0.1). (DOC)
