# Supplementary material for: Gene-Gene Associations with the Susceptibility of Kawasaki Disease and Coronary Artery Lesions
Source: PLoS One. 2015 Nov 30;10(11):e0143056. doi: 10.1371/journal.pone.0143056 (PMC4664466; doi:10.1371/journal.pone.0143056)
Supplement: S1 Table — (DOC) [file pone.0143056.s001.doc]

**S1 Table.** The SNPs excluded for the final analysis due to call rate < 90% or their genotypes’ distributions beyond Hardy Weinberg equilibrium (HWE) ≦ 0.001.

**a) 17 SNPs with call rate < 90%**

| **Gene** | **Chro Chr. position / dbSNP** | **Polymorphism** | **Call rate** |
| --- | --- | --- | --- |
| *ZNF595* | 4p16.3 / rs7667398 | C/T | 0 % |
| *TCF7* | 5q31.1 / rs5742913 | C/T | 50.7 % |
| *IL17F* | 6p12 / rs763780 | A/G | 50.0 % |
| *HLA-DQB1* | 6p21.3 / rs2300825 | A/G | 73.6 % |
| *HLA-DRB5* | 6p21.3 / rs6922050 | A/G | 75.5 % |
| *SOD2* | 6q25.3 / rs4880 | C/T | 79.5 % |
| *IL15RA* | 10p15-p14 / rs2296139 | A/G | 0 % |
| *CXCL12* | 10q11.1 / rs2297630 | A/G | 86.9 % |
| *ELF5* | 11p13-p12 / rs942223 | A/G | 83.2 % |
| *NOD2* | 16q21 / rs2066844 | C/T | 87.6 % |
| *ITGB3* | 17q21.32 / rs5918 | C/T | 0.5 % |
| *ITGB3* | 17q21.32 / rs2317676 | A/G | 71.0 % |
| *CLEC4M* | 19p13 / rs605673 | C/T | 82.6 % |
| *EMR2* | 19p13.1 / rs10418770 | A/G | 77.3 % |
| *ADAM33* | 20p13 / rs487377 | A/G | 89.7 % |
| *ADAM33* | 20p13 / rs528557 | C/G | 87.0 % |
| *MIF* | 22q11.23 / rs2070766 | C/G | 0 % |
| **b) 22 SNPs beyond HWE** | | | |
| Gene | **Chr. position / dbSNP** | **Polymorphism** | ***p* value** |
| *CHL1* | 3p26.1 / rs9681389 | C/G | = 0.001 |
| *TLR6* | 4p14 / rs5743810 | C/T | ≦ 0.001 |
| *ZNF595* | 4p16.3 / rs9328744 | A/G | ≦ 0.001 |
| *HLA-DQA1* | 6p21.3 / rs1061172 | A/G | ≦ 0.001 |
| *LTA* | 6p21.3 / rs2071590 | C/T | ≦ 0.001 |
| *TAP2* | 6p21.3 / rs241447 | A/G | ≦ 0.001 |
| *HLA-DQB1* | 6p21.3 / rs2854275 | G/T | ≦ 0.001 |
| *HLA-DRB1* | 6p21.3 / rs2760980 | A/G | ≦ 0.001 |
| *HLA-DRB1* | 6p21.3 / rs2395222 | A/G | ≦ 0.001 |
| *HLA-DRB1* | 6p21.3 / rs4713552 | C/T | ≦ 0.001 |
| *HLA-DRB1* | 6p21.3 / rs7753335 | A/C | ≦ 0.001 |
| *HLA-DRB1* | 6p21.3 / rs9269794 | C/G | ≦ 0.001 |
| *INPP5A* | 10q26.3 / rs1133400 | A/G | ≦ 0.001 |
| *NOD2* | 16q21 / rs748855 | A/G | ≦ 0.001 |
| *CCL3* | 17q11-q21 / rs1130371 | C/T | ≦ 0.001 |
| *CD97* | 19p13 / rs10421250 | A/C | ≦ 0.001 |
| *C3* | 19p13.3-p13.2 / rs344548 | C/G | ≦ 0.001 |
| *FOXP3* | Xp11.23 / rs3761548 | A/C | ≦ 0.001 |
| *FOXP3* | Xp11.23 / rs3761549 | C/T | ≦ 0.001 |
| *FOXP3* | Xp11.23 / rs2280883 | C/T | ≦ 0.001 |
| *TIMP1* | Xp11.3-p11.23 / rs4898 | C/T | ≦ 0.001 |
| *CXCR3* | Xq13 / rs2280964 | C/T | ≦ 0.001 |
