# Supplementary material for: Gene-Gene Associations with the Susceptibility of Kawasaki Disease and Coronary Artery Lesions
Source: PLoS One. 2015 Nov 30;10(11):e0143056. doi: 10.1371/journal.pone.0143056 (PMC4664466; doi:10.1371/journal.pone.0143056)
Supplement: S2 Table — (DOC) [file pone.0143056.s002.doc]

**S2 Table.** Association of 31 SNPs in 27 innate, adaptive and stress/response genes with CB and KD cohort UVA analysis (*p* < 0.1).

| **Gene** | **Chromosome position** | **Location** | **^a^MAF** | **^b^HWE** | ***p* value (UVA)** |
| --- | --- | --- | --- | --- | --- |
| Innate immunity |  |  |  |  |  |
| *COLEC11,* rs10210631 | 2p25.3 | Intron 3, A/G | 0.360 | 0.135 | 0.087 |
| *SPP1,* rs2728127 | 4q21-q25 | Promoter, A/G | 0.369 | 0.597 | 0.046 |
| *SPP1,* rs2853744 | 4q21-q25 | Promoter, G/T | 0.378 | 0.703 | 0.098 |
| *C5,* rs17611 | 9q33-q34 | Exon 19, A/G | 0.462 | 0.275 | 0.052 |
| *CXCL12,* rs266093 | 10q11.1 | 3' UTR, C/G | 0.131 | 0.015 | 0.014 |
| *CLEC4C,* rs10845821 | 12p13.2-p12.3 | Intron 1, C/T | 0.341 | 0.413 | 0.026 |
| *CD209,* rs2287886 | 19p13 | Promoter, A/G | 0.276 | 0.007 | 0.004 |
| Adaptive immunity |  |  |  |  |  |
| *LY75,* rs2042772 | 2q24 | Intron 34, C/T | 0.345 | 0.572 | 0.044 |
| *IL5RA,* rs340833 | 3p26-p24 | 3' UTR, A/G | 0.282 | 0.762 | 0.024 |
| *IL13,* rs1800925 | 5q31 | Promoter, C/T | 0.186 | 0.723 | 0.048 |
| *HLA-DPB1,* rs3097671 | 6p21.3 | Intron 1, C/G | 0.078 | 0.737 | 0.059 |
| *HLA-DQA1,* rs2040410 | 6p21.3 | Intergene, A/G | 0.080 | 0.020 | 0.046 |
| *TAP1,* rs2071541 | 6p21.3 | Promoter, C/T | 0.142 | 0.163 | 0.054 |
| *TAP2,* rs2071544 | 6p21.3 | Intron 1, A/G | 0.364 | 0.588 | 0.046 |
| *TBX21,* rs2240017 | 17q21.32 | Exon 1, C/G | 0.089 | 0.292 | 0.002 |
| *TGFB1,* rs1800469 | 19q13.1 | Promoter, C/T | 0.478 | 0.661 | 0.041 |
| Stress and response |  |  |  |  |  |
| *PIK3CD,* rs11121484 | 1p36.2 | Exon 22, C/T | 0.071 | 0.060 | 0.050 |
| *SELP,* rs6128 | 1q22-q25 | Exon 14, A/G | 0.345 | 0.365 | 0.078 |
| *PDGFRA,* rs4358459 | 4q11-q13 | Exon 7, G/T | 0.173 | 0.080 | 0.072 |
| *ADRB2,* rs1042713 | 5q31-q32 | Exon 1, A/G | 0.438 | 0.013 | 0.046 |
| *ADRB2,* rs6888329 | 5q31-q32 | Intergene, A/G | 0.365 | 0.408 | 0.096 |
| *CYFIP2,* rs767007 | 5q33.3 | Promoter, C/G | 0.491 | 0.509 | 0.034 |
| *PEX6,* rs2274514 | 6p21.1 | Intron 9, A/G | 0.215 | 0.530 | 0.073 |
| *PIM1,* rs262918 | 6p21.2 | Intergene, C/T | 0.325 | 0.137 | 0.050 |
| *PSMB8,* rs3763364 | 6p21.3 | 3' UTR, A/T | 0.469 | 0.647 | 0.029 |
| *NPSR1,* rs323922 | 7p14.3 | Intron 2, C/G | 0.467 | 0.316 | 0.066 |
| *NPSR1,* rs324377 | 7p14.3 | Intron 2, A/C | 0.462 | 0.210 | 0.082 |
| *ELF5,* rs836145 | 11p13-p12 | Intron 1, G/T | 0.436 | 0.271 | 0.058 |
| *PDE2A,* rs341058 | 11q13.4 | Promoter, A/G | 0.329 | 0.421 | 0.012 |
| *PDE2A,* rs458437 | 11q13.4 | Intron 1, G/T | 0.491 | 0.693 | 0.050 |
| *ADAM33,* rs3918400 | 20p13 | 3' UTR, C/T | 0.182 | 0.783 | 0.058 |

Notes: ^a^MAF = minor allele frequency; ^b^HWE = Hardy Weinberg equilibrium. Data including 575 CB and 226 KD dataset vs. 345 SNPs dataset were analyzed by univariate analysis.
