# Supplementary material for: Gene-Gene Associations with the Susceptibility of Kawasaki Disease and Coronary Artery Lesions
Source: PLoS One. 2015 Nov 30;10(11):e0143056. doi: 10.1371/journal.pone.0143056 (PMC4664466; doi:10.1371/journal.pone.0143056)
Supplement: S3 Table — (DOC) [file pone.0143056.s003.doc]

**S3 Table.** Association of 22 SNPs in 21 innate, adaptive and stress/response genes with/without CAL in KD cohort by UVA analysis (*p* < 0.1).

| **Gene** | **Chromosome position** | **Location** | **Risk genotype** | ***p* value**  **(UVA)** |
| --- | --- | --- | --- | --- |
| Innate immunity |  |  |  |  |
| *CCR5,* rs1799987 | 3p21.31 | Intron 1, A/G | AA | 0.055 |
| *CXCL10,* rs867562 | 4q21 | Intergene, A/G | AG | 0.090 |
| *CD14,* rs2569190 | 5q31.1 | Promoter, A/G | AA | 0.019 |
| *IL6,* rs1880242 | 7p21 | Intergene, G/T | TT | 0.094 |
| *CCL24,* rs2302004 | 7q11.23 | Intron 1, C/T | TC | 0.066 |
| *CLEC2D,* rs1863873 | 12p13 | Intergene, C/T | TC | 0.027 |
| *CLEC2D,* rs1863874 | 12p13 | Intergene, A/T | AT | 0.033 |
| *CLEC4C,* rs10845821 | 12p13.2-p12.3 | Intron 1, C/T | TT | 0.040 |
| *NOD2,* rs2111235 | 16q21 | Intron 2, C/T | CC | 0.023 |
| *CD209,* rs12611071 | 19p13 | Intergene, A/C | AA | 0.051 |
| Adaptive immunity |  |  |  |  |
| *LY75,* rs2042772 | 2q24 | Intron 34, C/T | TT | 0.011 |
| *CD80,* rs1485332 | 3q13.3-q21 | Intron 2, C/G | GG | 0.041 |
| *IL13,* rs1800925 | 5q31 | Promoter, C/T | TT | 0.078 |
| *IL4,* rs2243250 | 5q31.1 | Promoter, C/T | CC | 0.010 |
| *MS4A2,* rs2583476 | 11q13 | Intron 2, C/T | TC | 0.018 |
| *IL4R,* rs1805015 | 16p12.1-p11.2 | Exon 11, C/T | TC | 0.052 |
| Stress and response |  |  |  |  |
| *LTC4S,* rs730012 | 5q35 | Promoter, A/C | CC | 0.070 |
| *PEX6,* rs3763238 | 6p21.1 | Intergene, C/T | TC | 0.080 |
| *MSRA,* rs6984840 | 8p23.1 | Intron 3, A/G | GG | 0.092 |
| *EHF,* rs286902 | 11p12 | Intron 2, A/G | GG | 0.036 |
| *ITGB3,* rs3892085 | 17q21.32 | Intron 1, A/G | AG | 0.077 |
| *ADAM33,* rs3918400 | 20p13 | 3' UTR, C/T | TC | 0.023 |

Notes: Data including 73 cases with coronary artery lesions (CAL) and 153 cases without CAL between Kawasaki disease (KD) dataset vs. 345 SNPs dataset were analyzed by univariate analysis.
